# Access to and perceived unmet need for mental health services and support in a community sample of UK adolescents with and without experience of childhood adversity

**DOI:** 10.1017/S2045796024000027

**Published:** 2024-01-24

**Authors:** E. Soneson, S. R. White, E. Howarth, T. Ford, M. Fazel, P.B. Jones

**Affiliations:** 1 Department of Psychiatry, University of Oxford, Oxford, UK; 2 Department of Psychiatry, University of Cambridge, Cambridge, UK; 3 MRC Biostatistics Unit, University of Cambridge, UK; 4 School of Psychology, University of East London, London, UK

**Keywords:** mental health services, psychiatric services, adversity, adolescents, adverse childhood experiences, help-seeking

## Abstract

**Aims:**

Children and adolescents with a history of adverse childhood experiences (ACEs) are more likely than their peers to develop mental health difficulties, but not enough is known about their help-seeking behaviours and preferences. We aimed to determine whether and how ACEs are associated with access to and perceived unmet need for mental health services and support amongst secondary school students.

**Methods:**

We used multi-level logistic regression with data from the 2020 OxWell Student Survey to assess whether ACEs were associated with (1) prior access to mental health support and (2) perceived unmet need for mental health services in a community sample of English secondary school students. We assessed ACEs as a cumulative score from the Center for Youth Wellness Adverse Childhood Experiences Questionnaire: Teen Self-Report version and accounted for current mental health difficulties as measured by the 25-item Revised Children’s Anxiety and Depression Scale (RCADS).

**Results:**

Our analysis included 2018 students across 64 schools, of whom 29.9% (598/2002) reported prior access to mental health support and 34.1% (469/1377) reported a perceived unmet need for services. In the unadjusted models, the cumulative ACE score was significantly positively associated with both prior access to mental health support (odds ratio (OR)=1.36; 95% confidence interval (CI) 1.29–1.43) and perceived unmet need for mental health services (OR=1.47; 95% CI 1.37–1.59), meaning that students who had experienced adversity had a greater chance of having previously accessed support as well as perceiving an unmet need for services. After adjusting for mental health difficulties and other sociodemographic variables, cumulative ACE scores were positively associated with prior access (adjusted OR (aOR)=1.25; 95% CI 1.17–1.34 with a significant interaction between RCADS and ACE scores, aOR=0.88; 95% CI 0.84–0.93) as well as perceived unmet need (aOR=1.32; 95% CI 1.21–1.43 with a significant interaction between RCADS and ACE scores, aOR=0.85; 95% CI 0.78–0.91).

**Conclusions:**

Although it is encouraging that adolescents with experience of adversity are more likely than their peers with similar levels of depression and anxiety symptoms to have accessed mental health support, there remains a concern that those who *have not* accessed support are more likely to perceive an as-yet unmet need for it. Mental health support must be available, accessible, and acceptable to all who need it, especially for those groups that traditionally have not accessed services, including the more marginalised and vulnerable populations.

## Introduction

Early intervention may improve mental health outcomes, yet only one-quarter to one-half of children and adolescents with diagnosable disorders access mental health services (Ford et al., 2007; Merikangas et al., 2011). Several factors influence access, including characteristics of the presenting mental health difficulties, recognition of difficulties, knowledge of where to seek support, attitudes toward help-seeking, and perceptions of support (Radez et al., 2020). In addition, parents’ recognition of their child’s mental health difficulties, as well as their attitudes toward mental health, knowledge of available support, and perceptions of service providers and treatment options may influence their decisions to seek support on behalf of their child (Crouch et al., 2019; Reardon et al., 2017). At the systems level, access is influenced by factors such as service availability and flexibility, waiting times, cost, and administrative processes (Anderson et al., 2017). These multi-level barriers demonstrate the complex nature of accessing timely and effective mental health support.

One underexplored area is how experience of childhood adversity might impact access to mental health support. Research on childhood adversity has often focused on a ‘core set’ of adversities termed ‘adverse childhood experiences’ (ACEs), which comprise child maltreatment and household dysfunction. ACEs are common amongst children and adolescents (McLaughlin et al., 2012), and clear and consistent evidence has demonstrated a strong association between adversity and a range of poor mental health outcomes in childhood and adolescence (Dunn et al., 2011; McLaughlin et al., 2012). In a sample of US adolescents, McLaughlin et al. (2012) estimated that population-attributable risk proportions for a set of twelve ACEs ranged from 15.7% for fear disorders to 40.7% of behaviour disorders. As such, adversity represents a profound social determinant of mental health that requires urgent action and attention in terms of both prevention and intervention (Bhutta et al., 2023; Ceccarelli et al., 2022).

In terms of the possible influence of ACEs on mental health help-seeking, it is plausible that exposure could serve as a barrier. For example, experiences of maltreatment or family dysfunction could reduce an adolescent’s trust in adults and impede them from seeking support (Leavey et al., 2011; Lester et al., 2020; Munoz et al., 2019), or may signal that parents or carers are less willing or able to seek support on their child’s behalf (Leavey et al., 2011; Stebbins, 2020; Villagrana, 2010). Conversely, if recognised and acted upon by professionals, adversity could serve as a facilitator for accessing mental health services by bringing vulnerable children and adolescents to the attention of wider systems of care (e.g. schools, medical providers, or social care; Stebbins, 2020). Individuals in these systems may then facilitate access to support (Horwitz et al., 2012; Stebbins, 2020).

Whilst several studies have examined whether experience of adversity is associated with service use, most do not adjust for mental health difficulties, which complicates interpretation because the correlates of accessing support may actually be correlates of the mental health difficulties themselves (Ford, 2008). The few studies that have examined whether experience of adversity is *independently* related to service use have substantial methodological differences and report conflicting findings. For example, Alcalá and Balkrishnan (2019) used the US National Survey of Children’s Health to determine the individual and cumulative associations of nine ACEs with past-year service use for children and adolescents aged 2-17 years. Amongst those with a parent-reported lifetime ‘behavioural, mental, congenital, or developmental condition’ (20%; N=17,146), most adversities were significantly associated with an increased likelihood of service use (adjusted odds ratios (aORs)=1.23–2.27), as was the total number of ACEs (the ‘cumulative ACE score’; aOR=1.27; 95% confidence interval (CI) 1.21–1.32). However, a second analysis of data from the 2016 wave of the same survey did not replicate these findings: amongst those 6- to 17-year-olds whom parents identified as having a past-year ‘mental or behavioural condition’ requiring treatment or counselling (15%; N=5723), Stebbins (2020) reported no clear relationship between the cumulative ACE score and service use. A further analysis of the subsample of 3812 children and adolescents with any ACE demonstrated that increased parental vulnerability, including poor parental coping and low emotional support, reduced the likelihood of service use. After controlling for parental factors, those with two (aOR=0.47, 95% CI: 0.29, 0.76) or three (aOR=0.56, 95% CI: 0.33, 0.96), but not four or more, ACEs were significantly less likely than those with one ACE to have accessed services.

Another relevant consideration for understanding help-seeking pathways is perceived need, referring to beliefs about whether one would benefit from support. Children and adolescents who have a perceived need but who have not accessed support that adequately addresses this need (i.e. a ‘perceived unmet need’) are a particularly important group about whom little is known. A recent survey from a representative community sample of 2310 Australian adolescents aged 13-17 years provides some insight on this matter (Schnyder et al., 2019). Of the one-third who identified a need for support, around half reported that their needs were fully met (i.e. that they had accessed sufficient support), one-in-four reported that their needs were partially met (i.e. they had accessed some support but that it was not sufficient), and one-in-five reported that their needs were unmet (i.e. they needed support but had never accessed any). To our knowledge, however, no studies have quantitatively explored perceived (unmet) need amongst those who have experienced adversity.

Understanding how vulnerable groups interact with mental health services and support is critical for ensuring that all adolescents can access support when needed. In this study, we used data from a large school-based survey to determine whether and how experience of adversity is associated with access to and perceived unmet need for mental health services and support amongst secondary school students.

## Methods

### Data source: OxWell Student Survey

This study used data from the June-July 2020 wave of the OxWell Student Survey, a repeated cross-sectional, online, self-report survey of student mental health and wellbeing (Mansfield et al., 2021). The target study population for the survey consists of students aged 8-18 years in English primary and secondary schools and further education colleges (FECs). The study team recruits participating schools in partnership with local authorities. Schools then enrol their students by providing them with secure log-in details to an online platform where they can read about the survey and provide informed consent (ages 16+) before taking part.

The survey is typically administered during school day, but due to the Covid-19-related partial school closures, the study team adapted procedures to allow students to complete the 2020 survey from their homes if they were not receiving in-school educational provision. The survey platform does not collect any identifiable information about participants, which (1) helps increase the diversity and representativeness of the sample, (2) enables a parental opt-out model to ensure maximum participation, and (3) encourages more accurate and honest responses (Mansfield et al., 2021). The study was approved by the University of Oxford Research Ethics Committee (Reference: R62366/RE001).

We used the school identifier collected in the OxWell data to link student responses to publicly-available school- and area-level characteristics collected by the Office for National Statistics (ONS; Gov.uk, 2019) and Department for Education (DfE; Gov.uk, 2020).

### Participants

Participants in these analyses were adolescents in Years 12 and 13 (approx. age 16-18 years) at state-maintained and independent secondary schools and FECs located primarily in Oxfordshire, Berkshire, and Buckinghamshire.

### Measures

#### Access to mental health support and perceived unmet need for mental health services

The OxWell survey assessed prior access to mental health support with the question ‘Have you ever received any mental health support (e.g. from an adult at school or college/CAMHS/private psychologist/counsellor/charity service)?’ (‘yes’, ‘no’). Amongst those who *had not previously accessed mental health support*, the survey assessed perceived unmet need for mental health services with the question ‘Have you ever felt that you could have benefitted from using mental health services?’ (‘yes’, ‘no’).

#### Adverse childhood experiences

The survey measured cumulative ACE scores using the Center for Youth Wellness Adverse Childhood Experiences Questionnaire (CYW ACE-Q): Teen Self-Report version (Center for Youth Wellness, 2015). The questionnaire presents participants with a list of ten ACEs including child maltreatment (physical, sexual, and emotional abuse; physical and emotional neglect; and witnessing domestic violence) and household dysfunction (parental separation/divorce, drug or alcohol use of a household member, incarceration of a household member, and mental ill health of a household member) and asks them to indicate the number of statements that apply to them (i.e. cumulative scores range 0-10). As such, it does not give information on individual ACEs. Psychometric properties for the CYW ACE-Q have not been reported, but it is a recommended measure for assessing ACEs (Oh et al., 2018).

#### Covariates

The survey assessed mental health difficulties using the 25-item Revised Children’s Anxiety and Depression Scale (RCADS-25; Ebesutani et al., 2012). All statements have four response categories according to frequency (0 – ‘never’, 1 – ‘sometimes’, 2 – ‘often’, 3 – ‘always’). Responses sum to a Total Anxiety and Depression score (0-75), with higher scores indicating greater difficulties. In accordance with scoring guidance, we transformed these scores into t-scores by gender and age category and took the diagnostic threshold to be t-scores ≥ 70, (Chorpita, 2020). The RCADS-25 has demonstrated acceptable reliability in school-based samples and adequately discriminates between those with and without mental health diagnoses (Ebesutani et al., 2012).

The survey also includes the following sociodemographic variables, which we used as covariates: gender (‘girl’ or ‘boy’), school year (12 or 13), free school meal eligibility (‘yes’, ‘no’, ‘don’t know’), and birthplace (born in the UK; ‘yes’, ‘no’, ‘would rather not say’).

### Analysis

We used DfE and ONS data to explore the characteristics of the participating schools and compare with national statistics. For both outcomes (prior access and perceived unmet need), we calculated unadjusted ORs and 95% CIs for our main independent variable (cumulative ACE score) and covariates (RCADS score, gender, school year, free school meal eligibility, and student birthplace). In our main analyses, for the free school meal eligibility variable, we re-coded ‘don’t know’ responses as ‘no’ as a conservative estimate of eligibility, and for the birthplace variable, we re-coded ‘prefer not to say’ as missing data. We used complete-cases multi-level logistic regression (R *lme4* package; Bates et al., 2015) with school as a random effect to examine the relationship between cumulative ACE score and our two outcomes, adjusting for the covariates listed above and including an interaction term between cumulative ACE score and RCADS score. We additionally scaled the RCADS score to improve the models’ numerical stability.

## Results

### Participants

2360 Year 12 and 13 students across 65 secondary schools and FECs accessed the survey (N.B. an exact response rate is not possible under our recruitment model; Mansfield et al., 2021). Survey administrators removed 342 (14.5%) responses due to non-engagement or unrealistic/inconsistent responses (Mansfield et al., 2021), leaving a final sample of 2018 participants across 64 schools (Table 1).

Compared with national statistics, participating schools were slightly larger (mean headcount=1115 vs. 986), more ethnically diverse (mean percentage White British=59.7% vs. 66.7%). Participating schools had a lower percentage of students eligible for free school meals than the national secondary school average (mean percentage=8.3% vs. 17.3%), although it should be noted that nationally, the proportion of eligible students drops drastically in Years 12/13.

### Prior access to mental health support

**Figure 1:**
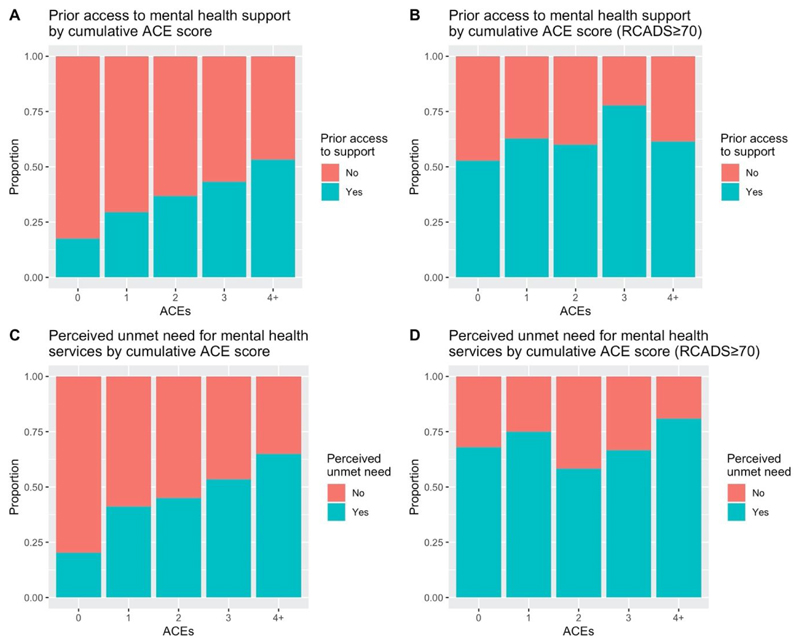
(A) Proportion of students with prior access to mental health support by cumulative ACE score for the total sample of Year 12/13 students (N = 1960); (B) Proportion of students with prior access to mental health support by cumulative ACE score for the 14% of students scoring in the clinical range on the RCADS (N = 271); (C) Proportion of students with perceived unmet need for mental health services by cumulative ACE score for the total sample of Year 12/13 students (N = 1321); (D) Proportion of students with perceived unmet need for mental health services by cumulative ACE score for the 8.1% of students scoring in the clinical range on the RCADS (N = 107).

598 of 2002 students (29.9%) reported prior access to mental health support (Table 2), including 61.6% (170/276) of those scoring above the clinical threshold on the RCADS and 24.5% (401/1639) of those scoring below. Figure 1 shows the proportion of students who had previously accessed mental health support by cumulative ACE score in the full sample (Figure 1A) as well as those scoring above the RCADS clinical threshold (Figure 1B).

In the unadjusted model, the cumulative ACE score was significantly positively associated with prior access to mental health support (OR=1.36; 95% CI 1.29–1.43), meaning that students who had experienced adversity had a greater likelihood of having previously accessed support. Complete cases analysis resulted in a final sample size of N=1867 (93.3% of those with outcome data; Table 2), with exclusion from the model due primarily to missingness on RCADS scores (N=87) and cumulative ACE scores (N=42; Supplementary Table 1). Figure 1 indicates a potential interaction between ACEs and RCADS, as shown by visually different patterns between Figures 1A and 1B. This interaction was significant (aOR=0.88; 95% CI 0.84–0.93; see Supplementary Figure 1 for the modelled proportion accounting for this interaction). The cumulative ACE score was positively associated with access to support (for a ‘typical’ student at a ‘typical’ school, with an average RCADS score, aOR=1.25; 95% CI 1.17–1.34).

### Perceived unmet need for mental health services

469 of 1377 students (34.1%) perceived an unmet need for mental health services (Table 3), including 72.0% (77/107) of those scoring above the clinical threshold on the RCADS as well as 22.9% (278/1214) of those scoring below. Figure 1 shows the proportion of students with a perceived unmet need for mental health services by cumulative ACE score in the full sample (Figure 1C) and for those scoring above the RCADS clinical threshold (Figure 1D).

In the unadjusted model, the cumulative ACE score was significantly positively associated with perceived unmet need for mental health services (OR=1.47; 95% CI 1.37–1.59), meaning that students who had experienced adversity had a greater likelihood of perceiving an unmet need for mental health services. Complete cases analysis resulted in a final sample size of N=1284 (93.2% of those with outcome data; Table 3), with exclusion from the model due primarily to missingness on RCADS scores (N=56) and cumulative ACE scores (N=31; Supplementary Table 1). Figure 1 indicates a potential interaction between ACEs and RCADS, as shown by visually different patterns between Figures 1C and 1D. This interaction was significant (aOR=0.85; 95% CI 0.78–0.91; see Supplementary Figure 2 for the modelled proportion accounting for this interaction). The cumulative ACE score was positively associated with perceived unmet need for services (for a ‘typical’ student, aOR=1.32; 95% CI 1.21–1.43).

## Discussion

We analysed survey data from a community sample of school-aged students to explore mental health help-seeking amongst adolescents with self-reported experience of childhood adversity. Both before and after adjusting for current mental health difficulties, experience of adversity (measured as a cumulative ACE score) was associated with a greater likelihood of prior access to mental health support and perceived unmet need for mental health services.

In the context of the mixed evidence, it was encouraging that experience of adversity was associated with a higher likelihood of prior access to support. One possible explanation relates to systems of care – the coordinated networks of community-based services and supports that can help facilitate access to mental health support if and when needed (Miller et al., 2012; Stebbins, 2020). Whilst parents are often acknowledged as the primary gatekeepers for their children’s mental health (Crouch et al., 2019; Radez et al., 2021; Reardon et al., 2017; Ryan et al., 2015), there are many other individuals with a similar role, including social workers, mental health professionals, other health and allied health professionals, school staff, and criminal justice professionals (Duong et al., 2021; Stebbins, 2020). Knowledge of adversity may trigger these individuals to identify difficulties and encourage help-seeking. For example, Horwitz and colleagues (2012) have shown that as children with possible experience of maltreatment come into contact with schools or medical professionals, or have more intensive involvement with the child welfare system, they are more likely to use mental health services.

Our findings additionally offer insight into perceived unmet need for mental health services, an important indicator of service availability, accessibility, and acceptability. Whilst others have studied this concept in the general adolescent population (Schnyder et al., 2019), our study provides unique insight within the context of adversity. It is concerning that those who have experienced adversity were more likely than their peers with similar levels of mental health difficulties to perceive an unmet need, and it is important to understand why these adolescents are not accessing support when they feel as though they could benefit from it. It may be that they have more complex, multi-faceted needs in addition to their mental health needs (Kerns et al., 2014; Lester et al., 2020), or face additional or amplified barriers in comparison with their peers, such as concerns about confidentiality or parental involvement (Lester et al., 2020; Soleimanpour et al., 2017). They might also struggle to trust adults due to their specific experiences, which could prevent them from seeking or engaging with help (Kerns et al., 2014; Lester et al., 2020; Munoz et al., 2019).

### Limitations

This study has several strengths, including use of a large community sample of adolescents, consideration of multiple aspects of the help-seeking pathway, and use of statistical models that consider the multi-level structure of the data. However, there are also a number of limitations. In terms of the sample, whilst the schools recruited were diverse in their sociodemographic characteristics, determining sample representativeness is difficult because characteristics such as ethnicity, non-binary gender identities, and special educational needs are not assessed in the survey. Furthermore, the participation rate for 2020 will have been lower than in previous waves because of school closures. This has important implications: students in unsafe home environments may not have accessed the survey or may have done so but provided inaccurate responses (for example, if a parent was present).

In terms of the survey data, the self-report nature may have introduced bias into our assessment of prior access to mental health support, as there may be discrepancies between participants’ reports and, for example, contact recorded in administrative health records. Conversely, self-report may have captured additional contacts not routinely recorded. There was also a discrepancy in the wording of our outcome measures: whilst the question about prior access asked about *mental health support*, the question about perceived unmet need asked about *mental health services*. Students may have interpreted these concepts differently, which may have led to an underestimation of the proportion reporting a perceived unmet need. Furthermore, if students responded that they had previously accessed support, they were not asked about perceived need, which will also have led to an underestimation, as students who have previously accessed support might still perceive a need for different or additional support. For ACEs, the survey collected only a cumulative score, without information about individual ACEs, timing, severity, or chronicity. Although this helps maintain anonymity and might give students greater confidence to disclose adversity, cumulative scores have limitations (Lacey & Minnis, 2019). Furthermore, a substantial evidence base demonstrates that retrospective reports of ACEs often poorly align with prospectively-ascertained reports (Baldwin et al., 2019; Danese, 2020), which may introduce bias and influence the interpretation and implications of findings.

Finally, the cross-sectional nature of the data presents challenges for interpretation, as we were not able to determine when ACEs occurred. To better understand how experience of adversity influences access to mental health support, we would need to focus on the students who experienced adversity *before* attempting to access support. Furthermore, these data do not allow us to assess mental health *at the time of accessing support*. Mental health is dynamic, and students who had accessed helpful mental health support would potentially have lower RCADS scores at the time of completing the survey than before accessing that support, which would have influenced our effect estimates.

### Implications for practice

Given concerns that vulnerable children and adolescents may have reduced access to mental health services, it was encouraging to find that those with experience of adversity were more likely to have accessed support than their peers with similar levels of current mental health difficulties. However, their increased likelihood of perceiving an unmet need highlights the possible additional needs of this group and indicates that different structures might be needed in order to improve access (Soleimanpour et al., 2017). There are many potential reasons why these adolescents may not have accessed support. For some, support may not have been available, whilst for others, support may have been available but not accessible. Still others may not have sought support due to negative perceptions of it or concerns about confidentiality and privacy (Soleimanpour et al., 2017; Yap et al., 2013). Engaging with adolescents and their families is essential for improving services (Fazel & Hoagwood, 2021; Lester et al., 2020), especially given their insights into the barriers and facilitators of accessing and remaining engaged with the current systems (Radez et al., 2020, 2021; Reardon et al., 2017).

### Implications for future research

Longitudinal data that allow for the establishment of chronology (i.e. relative timing of ACEs and access to services) could be an important next step in understanding pathways to support. Future studies could also move beyond a cumulative ACE score to understand the effects of individual ACEs as well as their timing, chronicity, and severity (McLaughlin, 2016). Considering how ACEs cluster is also likely to be important (Lacey, 2023), as this may have implications for tailoring services and identifying which types of services may be most effective and acceptable for these individuals (Lacey & Minnis, 2019). There is also scope to explore barriers to accessing support amongst those with a perceived unmet need: whilst many have studied barriers for the general adolescent population (see Radez et al., 2020 for a review) and some have examined barriers for children in care (Kerns et al., 2014), little research has focused on the specific barriers for the broader population of adolescents who have experienced adversity.

## Conclusions

Although it is encouraging that adolescents with experience of adversity are more likely than their peers with similar levels of current depression and anxiety symptoms to have accessed mental health support, there remains a concern that those who have not accessed support are more likely to perceive an unmet need for it. These adolescents may have more complex, multi-faceted needs in addition to their mental health needs or face additional or amplified barriers to accessing support. Mental health support must be available, accessible, and acceptable to all who need it, especially for marginalised and vulnerable populations.

## Supplementary Material

Supplementary Materials

## Figures and Tables

**Table 1:** Characteristics of participating schools (N = 57)^a^

Characteristic	
**School context**	
Urbanicity *N (%)*	
Rural	5 (8.8)
Urban	52 (91.2)
Area-level deprivation (Index of Multiple Deprivation) *Median (IQR)*	7 (4)
**Characteristics of school community**	
Percentage of students eligible for free school meals *Mean (SD)*	8.3 (5.6)
Percentage of students who are White British *Mean (SD)*	59.7 (26.6)
**Operational features of the school**	
Type of school *N (%)*	
State-funded	53 (93.0)
Independent	4 (7.0)
Mixed or single sex school *N (%)*	
Mixed	44 (77.2)
Girls only	7 (12.3)
Boys only	6 (10.5)
Number of students *Mean (SD)*	1115 (295)

a Data only available for N = 57 schools with linkable DfE and ONS data; all percentages use a denominator of 57. Characteristics of the school community are reported for N = 53 state-funded schools as independent schools are not required to report these.

b In the Index of Multiple Deprivation (McLennan et al., 2019), lower deciles represent relatively more deprived areas.

**Table 2:** Cumulative ACE score and prior access to mental health support

Prior access to mental health support							
	Total sample^a^	Have not accessed	Have accessed	Unadjusted OR	p-value	Adjusted OR N = 1867	p-value
	N=2002	N = 1404 (70.1%)	N = 598 (29.9%)	[95% CI]		[95% CI]	
**Individual-level factors**							
**Gender** *N* *(%)*							
Male	786	620	166	Ref.	Ref.	Ref.	Ref.
	(39.4)	(78.9)	(21.1)				
Female	1207	781	426	2.04	p<0.00	1.46	p=0.00
	(60.6)	(64.7)	(35.3)	[1.66,2.51]	1	[1.13,1.88]	4
**School year** *N (%)*							
Year 12	1635 (81.7)	1182 (72.3)	453 (27.7)	Ref.		Ref.	
Year 13	367 (18.3)	222 (60.5)	145 (39.5)	1.70 [1.34,2.16]	p<0.00 1	1.67 [1.27,2.21]	p<0.00 1
							
**Free school meals** ***N (%)***							
No	1661 (83.5)	1174 (70.7)	487 (29.3)	Ref.	Ref.	Ref.	Ref
Yes	123 (6.2)	73 (59.3)	50 (40.7)	1.65 [1.13,2.40]	p=0.01 0	1.26 [0.81,1.97]	p=0.29 8
Don’t	205 (10.3)	148 (72.2)	57 (27.8)	0.93 [0.67,1.28]	P=0.65 9	–	–
**Birthplace *N (%)***							
UK	1685 (84.2)	1158 (68.7)	527 (31.3)	Ref.	Ref.	Ref.	Ref.
Elsewhere	310 (15.5)	240 (77.4)	70 (22.6)	0.64 [0.48,0.85]	p=0.00 2	0.66 [0.47,0.92]	p=0.01 5
Prefer not to say	7 (0.4)	6 (85.7)	1 (14.3)	0.41 [0.02,2.49]	p=0.37 6	–	–
**Adverse childhood experiences**							
**Cumulative ACE score** *Median (IQR)*	1 (2)	0 (2)	2 (3.75)	1.36 [1.29,1.43]	p<0.00 1	1.25 [1.17,1.34]	p<0.00 1
**Mental health**							
**RCADS Total Score** *Mean (SD)*	51.1 (16.9)	47.2 (15.0)	60.5 (17.5)	1.05 [1.04,1.06]	p<0.00 1	–	–
**RCADS Total Score** (scaled)	–	–	–	–	–	2.51 [2.12,2.97]	p<0.00 1
**Interaction term**							
**RCADS Total Score** (scaled) * **Cumulative** **ACE score**	–	–	–	–	–	0.88 [0.84,0.93]	p<0.00 1

a Percentages are calculated using those who responded as a denominator, so Ns<2002 for each variable are due to missing data.

**Table 3:** Cumulative ACE score and perceived unmet need for mental health services

Perceived unmet need for mental health services							
	Total sample^a^	No perceive d unmet need	Perceive d unmet need	Unadjuste d OR	p-value	Adjusted OR N=1284	p-value
	N=1377	N = 908 (65.9%)	N=469 (34.1%)	[95% CI]		[95% CI]	
**Individual-level factors**							
**Gender *N (%)***							
Male	612 (44.5)	447 (73.0)	165 (27.0)	Ref.	Ref.	Ref.	Ref.
Female	763 (55.5)	461 (60.4)	302 (39.6)	1.77 [1.41,2.24]	p<0.00 1	1.27 [0.96,1.69]	p=0.09 9
**School year *N(%)***							
Year 12	1156 (84.0)	777 (67.2)	379 (32.8)	Ref.	Ref.	Ref.	Ref.
Year 13	221 (16.0)	131 (59.3)	90 (40.7)	1.41 [1.05,1.89]	p=0.02 4	1.51 [1.06,2.17]	p=0.02 4
**Free school meals *N (%)***							
No	1151 (84.1)	755 (65.6)	396 (34.4)	Ref.	Ref.	Ref.	Ref.
Yes	70 (5.1)	52 (74.3)	18 (25.7)	0.66 [0.37,1.13]	p=0.13 5	0.46 [0.24,0.88]	p=0.01 9
Don’t know	147 (10.7)	95 (64.6)	52 (35.4)	1.04 [0.72,1.49]	p=0.81 1	–	–
**Birthplace *N (%)***							
UK	1137 (82.6)	739 (65.0)	398 (35.0)	Ref.	Ref.	Ref.	Ref.
Elsewhere	234 (17.0)	163 (69.7)	71 (30.3)	0.81 [0.59,1.09]	p=0.17 1	0.75 [0.52,1.09]	p=0.13 1
Prefer not to say	6 (0.4)	6 (100)	0 (0.0)	N/A	–	–	–
**Adverse childhood experiences**							
**Cumulative** **ACE score** *Median (IQR)*	0 (2)	0 (1)	1 (3)	1.47 [1.37,1.59]	p<0.00 1	1.32 [1.21,1.43]	p<0.00 1
**Mental health**							
**RCADS Total Score** *Mean (SD)*	47.3 (15.1)	42.8 (12.4)	56.0 (16.0)	1.07 [1.06,1.08]	p<0.00 1	–	–
**RCADS Total** **Score** (scaled)	–	–	–	–	–	3.34 [2.68,4.18]	p<0.00
**Interaction term**							
**RCADS Total Score** (scaled) * Cumulative ACE score	–	–	–	–	–	0.85 [0.78,0.91]	p<0.00

a Percentages are calculated using those who responded as a denominator, so Ns<1377 for each variable are due to missing data. *N*.*B*. Only those who have not previously accessed support are presented with this question.

## Data Availability

The data used in this study are not publicly available. The study protocol and variable guides are available through the Open Science Framework (https://osf.io/sekhr/). The code for this analysis is available at https://github.com/OxWellStudy/ACEs-helpseeking-2023.
