# Supplementary material for: Access to and perceived unmet need for mental health services and support in a community sample of UK adolescents with and without experience of childhood adversity
Source: Epidemiol Psychiatr Sci. Author manuscript; Available in PMC 2024 Feb 14. (PMC7615639; doi:10.1017/S2045796024000027)
Supplement: Supplementary Materials [file EMS193241-supplement-Supplementary_Materials.pdf]

Supplementary Table 1. Comparison of students included and excluded from complete cases analyses

	Total sample	Prior access to mental health support			Perceived unmet need for mental health services		
		Subsample with outcome data	Included in complete cases analysis	Excluded from complete cases analysis	Subsample with outcome data[a]	Included in complete cases analysis	Excluded from complete cases analysis
	N = 2018	N = 2002	N = 1867	N = 135	N = 1377	N = 1284	N = 93
**Individual-level factors**							
**Gender** *N (%)*							
Male	793 (39.3)	786 (39.3)	735 (39.4)	51 (37.8)	612 (44.4)	573 (44.6)	39 (41.9)
Female	1216 (60.3)	1207 (60.3)	1132 (60.6)	75 (55.6)	763 (55.4)	711 (55.4)	52 (55.9)
Missing	9 (0.5)	9 (0.5)	0	9 (6.7)	2 (0.2)	0	2 (2.2)
**School year** *N (%)*							
Year 12	1647 (81.6)	1635 (81.7)	1528 (81.8)	107 (79.3)	1156 (84.0)	1083 (84.3)	73 (78.5)
Year 13	371 (18.4)	367 (18.3)	339 (18.2)	28 (20.7)	221 (16.0)	201 (15.7)	20 (21.5)
Missing	0	0	0	0	0	0	0
**Free school meals** *N (%)*							
No	1672 (82.9)	1661 (83.0)	1565 (83.8)	96 (71.1)	1151 (83.6)	1083 (84.3)	68 (73.1)
Yes	124 (6.1)	123 (6.1)	114 (6.1)	9 (6.7)	70 (5.1)	65 (5.1)	5 (5.4)
Don't know	209 (10.4)	205 (10.2)	188 (10.1)	17 (12.6)	147 (10.7)	136 (10.6)	11 (11.8)
Missing	13 (0.6)	13 (0.7)	0	13 (9.6)	9 (0.7)	0	9 (9.7)

	Total sample	Prior access to mental health support			Perceived unmet need for mental health services		
		Subsample with outcome data	Included in complete cases analysis	Excluded from complete cases analysis	Subsample with outcome data[a]	Included in complete cases analysis	Excluded from complete cases analysis
	N = 2018	N = 2002	N = 1867	N = 135	N = 1377	N = 1284	N = 93
Birthplace *N (%)*							
UK	1697 (84.1)	1685 (84.2)	1594 (85.4)	91 (67.4)	1137 (82.6)	1075 (83.7)	62 (66.7)
Elsewhere	313 (15.5)	310 (15.5)	273 (14.6)	37 (27.4)	234 (17.0)	209 (16.3)	25 (26.9)
Prefer not to say	8 (0.4)	7 (0.4)	0	7 (5.2)	6 (0.4)	0	6 (6.5)
Missing	0	0	0	0	0	0	0
**Adverse childhood experiences**							
Cumulative ACE score *Median (IQR)*	1 (2)	1 (2)	1 (2)	1 (2)	0 (2)	0 (2)	0 (1.75)
Missing *N (%)*	46 (2.3)	42 (2.1)	0	42 (31.1)	31 (2.3)	0	31 (33.3)
**Mental health**							
RCADS Total Score *Mean (SD)*	51.1 (16.9)	51.1 (16.9)	51.2 (16.9)	48.8 (18.3)	47.3 (15.1)	47.4 (15.1)	45.3 (16.3)
Missing *N (%)*	103 (5.1)	87 (4.3)	0	87 (64.4)	56 (4.1)	0	56 (60.2)

[a] Students are only presented with this question if they indicate that they have not previously accessed mental health support.

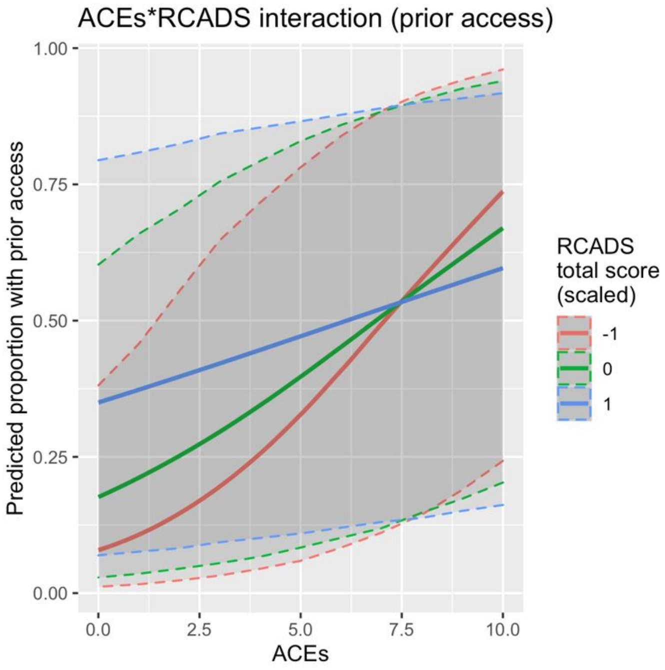

Supplementary Figure 1. Plot illustrating the interaction between number of ACEs and the scaled-RCADS total score for the prior access to mental health support. The plot shows how access to support varies across ACEs (0-10) and standard deviations of the scaled-RCADS score (-1, 0, and 1, where 0 is the mean, -1 is better mental health, and 1 is poorer mental health).

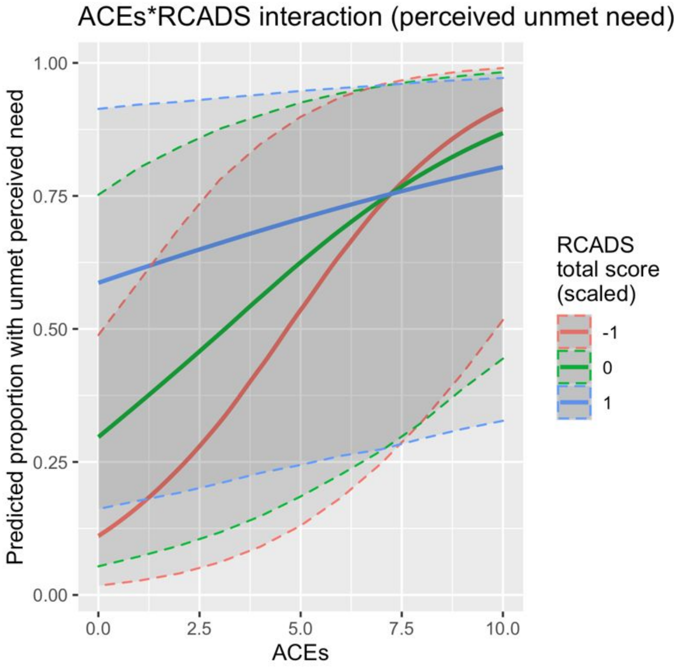

Supplementary Figure 2. Plot illustrating the interaction between number of ACEs and the scaled-RCADS total score for perceived unmet need for mental health services. The plot shows how perceived unmet need for mental health services varies across ACEs (0-10) and standard deviations of the scaled-RCADS score (-1, 0, and 1, where 0 is the mean, -1 is better mental health, and 1 is poorer mental health).
