# Supplementary material for: Access to and perceived unmet need for mental health services and support in a community sample of UK adolescents with and without experience of childhood adversity
Source: Epidemiol Psychiatr Sci. 2024 Jan 24;33:e1. doi: 10.1017/S2045796024000027 (PMC7615639; doi:10.1017/S2045796024000027)
Supplement: Supplementary file 1 [file S2045796024000027sup001.docx]

**Supplementary Figure 1.** Plot illustrating the interaction between number of ACEs and the scaled-RCADS total score for the prior access to mental health support. The plot shows how access to support varies across ACEs (0-10) and standard deviations of the scaled-RCADS score (-1, 0, and 1, where 0 is the mean, -1 is better mental health, and 1 is poorer mental health). 

**Supplementary Figure 2.** Plot illustrating the interaction between number of ACEs and the scaled-RCADS total score for perceived unmet need for mental health services. The plot shows how perceived unmet need for mental health services varies across ACEs (0-10) and standard deviations of the scaled-RCADS score (-1, 0, and 1, where 0 is the mean, -1 is better mental health, and 1 is poorer mental health).
