# Supplementary material for: Access to and perceived unmet need for mental health services and support in a community sample of UK adolescents with and without experience of childhood adversity
Source: Epidemiol Psychiatr Sci. 2024 Jan 24;33:e1. doi: 10.1017/S2045796024000027 (PMC7615639; doi:10.1017/S2045796024000027)
Supplement: Supplementary file 2 [file S2045796024000027sup002.docx]

**Supplementary Table 1.** Comparison of students included and excluded from complete cases analyses 

|  | Total sample | Prior access to mental health support |  |  | Perceived unmet need for mental health services |  |  |
| --- | --- | --- | --- | --- | --- | --- | --- |
|  |  | Subsample with outcome data | Included in complete cases analysis | Excluded from complete cases analysis | Subsample with outcome data^a^ | Included in complete cases analysis | Excluded from complete cases analysis |
|  | N = 2018 | N = 2002 | N = 1867 | N = 135 | N = 1377 | N = 1284 | N = 93 |
| Individual-level factors |  |  |  |  |  |  |  |
| Gender *N (%)* |  |  |  |  |  |  |  |
| Male | 793 (39.3) | 786 (39.3) | 735 (39.4) | 51 (37.8) | 612 (44.4) | 573 (44.6) | 39 (41.9) |
| Female | 1216 (60.3) | 1207 (60.3) | 1132 (60.6) | 75 (55.6) | 763 (55.4) | 711 (55.4) | 52 (55.9) |
| Missing | 9 (0.5) | 9 (0.5) | 0 | 9 (6.7) | 2 (0.2) | 0 | 2 (2.2) |
| School year *N (%)* |  |  |  |  |  |  |  |
| Year 12 | 1647 (81.6) | 1635 (81.7) | 1528 (81.8) | 107 (79.3) | 1156 (84.0) | 1083 (84.3) | 73 (78.5) |
| Year 13 | 371 (18.4) | 367 (18.3) | 339 (18.2) | 28 (20.7) | 221 (16.0) | 201 (15.7) | 20 (21.5) |
| Missing | 0 | 0 | 0 | 0 | 0 | 0 | 0 |
| Free school meals *N (%)* |  |  |  |  |  |  |  |
| No | 1672 (82.9) | 1661 (83.0) | 1565 (83.8) | 96 (71.1) | 1151 (83.6) | 1083 (84.3) | 68 (73.1) |
| Yes | 124 (6.1) | 123 (6.1) | 114 (6.1) | 9 (6.7) | 70 (5.1) | 65 (5.1) | 5 (5.4) |
| Don’t know | 209 (10.4) | 205 (10.2) | 188 (10.1) | 17 (12.6) | 147 (10.7) | 136 (10.6) | 11 (11.8) |
| Missing | 13 (0.6) | 13 (0.7) | 0 | 13 (9.6) | 9 (0.7) | 0 | 9 (9.7) |
| Birthplace *N (%)* |  |  |  |  |  |  |  |
| UK | 1697 (84.1) | 1685 (84.2) | 1594 (85.4) | 91 (67.4) | 1137 (82.6) | 1075 (83.7) | 62 (66.7) |
| Elsewhere | 313 (15.5) | 310 (15.5) | 273 (14.6) | 37 (27.4) | 234 (17.0) | 209 (16.3) | 25 (26.9) |
| Prefer not to say | 8 (0.4) | 7 (0.4) | 0 | 7 (5.2) | 6 (0.4) | 0 | 6 (6.5) |
| Missing | 0 | 0 | 0 | 0 | 0 | 0 | 0 |
| Adverse childhood experiences |  |  |  |  |  |  |  |
| Cumulative ACE score *Median (IQR)* | 1 (2) | 1 (2) | 1 (2) | 1 (2) | 0 (2) | 0 (2) | 0 (1.75) |
| Missing *N (%)* | 46 (2.3) | 42 (2.1) | 0 | 42 (31.1) | 31 (2.3) | 0 | 31 (33.3) |
| Mental health |  |  |  |  |  |  |  |
| RCADS Total Score *Mean (SD)* | 51.1 (16.9) | 51.1 (16.9) | 51.2 (16.9) | 48.8 (18.3) | 47.3 (15.1) | 47.4 (15.1) | 45.3 (16.3) |
| Missing *N (%)* | 103 (5.1) | 87 (4.3) | 0 | 87 (64.4) | 56 (4.1) | 0 | 56 (60.2) |

^a  ^Students are only presented with this question if they indicate that they have not previously accessed mental health support.
